# Isolation and Pharmacological Characterization of α-Elapitoxin-Ot1a, a Short-Chain Postsynaptic Neurotoxin from the Venom of the Western Desert Taipan, *Oxyuranus temporalis*

**DOI:** 10.3390/toxins8030058

**Published:** 2016-02-29

**Authors:** Carmel M. Barber, Muhamad Rusdi Ahmad Rusmili, Wayne C. Hodgson

**Affiliations:** 1 Monash Venom Group, Department of Pharmacology, Monash University, Clayton, VIC 3168, Australia; carmel.barber@gmail.com; 2 Department of Basic Medical Sciences, Kulliyyah of Pharmacy, International Islamic University Malaysia, Bandar Indera Mahkota 23800, Malaysia; rusdirusmili@iium.edu.my

**Keywords:** α-Elapitoxin-Ot1a, *Oxyuranus temporalis*, postsynaptic neurotoxin, antivenom, snake

## Abstract

Taipans (*Oxyuranus* spp.) are elapids with highly potent venoms containing presynaptic (β) and postsynaptic (α) neurotoxins. *O. temporalis* (Western Desert taipan), a newly discovered member of this genus, has been shown to possess venom which displays marked *in vitro* neurotoxicity. No components have been isolated from this venom. We describe the characterization of α-elapitoxin-Ot1a (α-EPTX-Ot1a; 6712 Da), a short-chain postsynaptic neurotoxin, which accounts for approximately 30% of *O. temporalis* venom. α-Elapitoxin-Ot1a (0.1–1 µM) produced concentration-dependent inhibition of indirect-twitches, and abolished contractile responses to exogenous acetylcholine and carbachol, in the chick biventer cervicis nerve-muscle preparation. The inhibition of indirect twitches by α-elapitoxin-Ot1a (1 µM) was not reversed by washing the tissue. Prior addition of taipan antivenom (10 U/mL) delayed the neurotoxic effects of α-elapitoxin-Ot1a (1 µM) and markedly attenuated the neurotoxic effects of α-elapitoxin-Ot1a (0.1 µM). α-Elapitoxin-Ot1a displayed pseudo-irreversible antagonism of concentration-response curves to carbachol with a pA_2_ value of 8.02 ± 0.05. *De novo* sequencing revealed the main sequence of the short-chain postsynaptic neurotoxin (*i.e.*, α-elapitoxin-Ot1a) as well as three other isoforms found in *O. temporalis* venom. α-Elapitoxin-Ot1a shows high sequence similarity (*i.e.*, >87%) with other taipan short-chain postsynaptic neurotoxins.

## 1. Introduction

The *Oxyuranus* genus consists of three species of highly venomous Australo-Papuan elapids; *i.e.*, inland taipan (*O. microlepidotus*), coastal taipan (*O. scutellatus*; found in Australia and Papua New Guinea) and the more recently discovered *O. temporalis* (Western Desert taipan, [1]). Due to the remote location of *O. temporalis*, only a handful of specimens have been caught and, as such, limited information exists about this species [1,2,3]. We recently showed that *O. temporalis* venom displays marked post-synaptic neurotoxic activity in isolated skeletal muscle [4].

Typically taipan venoms contain presynaptic and postsynaptic neurotoxins and have also been shown to contain natriuretic-like peptides [5,6], prothrombin activators [6,7,8,9], reversible calcium channels blockers (*i.e.*, taicatoxin, [6,10]), cysteine-rich secretory proteins (CRISP) [6] and Kunitz-type plasma kallikrein inhibitors [6,11]. The presynaptic neurotoxins isolated from taipan venoms are paradoxin (*O. microlepidotus*, [12]), taipoxin (*O. scutellatus*, [13]) and cannitoxin (*O. s. canni*, [14]), each consists of three subunits (α, β and γ) with molecular masses between 45 and 47 kDa [12,13,14,15]. A number of postsynaptic neurotoxins have been isolated from taipan venoms including; oxylepitoxin-1 (*O. microlepidotus*, [16]), α-scutoxin 1 (*O. scutellatus*, [17]), α-oxytoxin 1 (*O. s. canni*, [17]), taipan toxin 1 (*O. scutellatus*, [18]) and taipan toxin 2 (*O. scutellatus*, [18]). These short-chain neurotoxins have molecular masses between 6726–6789 Da. Many of these postsynaptic neurotoxins have also been pharmacologically characterized *in vitro* using the chick biventer cervicis nerve-muscle preparation, with an examination of their neurotoxicity and reversibility, as well as determination of potency (*i.e.*, pA_2_ values) and the effectiveness of antivenom in preventing their effects (as reviewed in [19]).

The aim of the present study was to isolate and pharmacologically characterize the major short-chain postsynaptic neurotoxin from *O. temporalis* venom.

## 2. Results

### 2.1. Fractionation of Venom via Reverse-Phase HPLC

**Figure 1:**
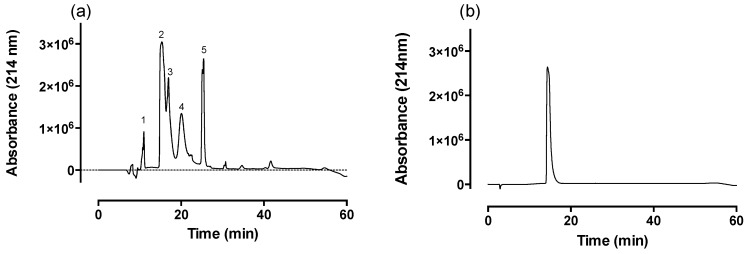
RP-HPLC chromatograph of (**a**) *O. temporalis* venom run on a Jupiter semi preparative C18 column and (**b**) α-elapitoxin-Ot1a run on a Jupiter analytical C18 column.

Fractionation of *O. temporalis* venom using a Jupiter semi preparative C18 column yielded five major peaks and a number of minor peaks (Figure 1a). Peak two, eluting around 15 min, showed marked postsynaptic neurotoxicity in the chick biventer cervicis nerve-muscle preparation. This peak was chosen for further analysis. α-Elapitoxin-Ot1a (peak 2) was collected and purified using an analytical C18 column, where α-elapitoxin-Ot1a eluted as a single peak around approximately 15 min (Figure 1b). α-Elapitoxin-Ot1a (and its isoforms) was found to make up 30.1% of *O. temporalis* venom based on the area under the curve of the HPLC profile.

### 2.2. Intact Protein Analysis with MALDI-TOF Mass Spectrometry

**Figure 2:**
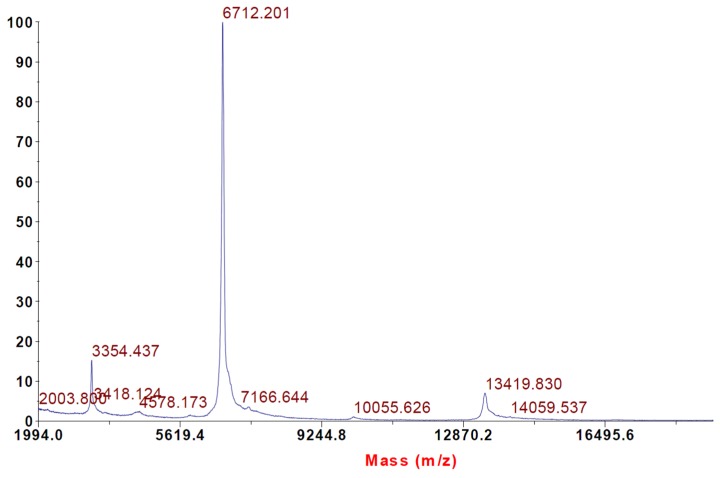
MALDI-TOF of α-elapitoxin-Ot1a indicating a molecular weight of 6712 Da. Proteins were analysed in Linear mode with a mass range of 5 kDa to 120 kDa.

Intact protein analysis of α-elapitoxin-Ot1a using MALDI-TOF showed the molecular weight to be 6712 Da (Figure 2).

### 2.3. Identification and de Novo Sequencing by LCMS/MS

Protein identification and *de novo* sequencing with PEAKS Studio 7 software (Version 7.0, Bioinformatics Solution Inc., Waterloo, ON, Canada, 2014) generated the following sequence for α-elapitoxin-Ot1a:

MTCYNQQSSQ AKTTTTCSGG VSSCYRKTWS DTRGTIIERG CGCPSVKKGI ERICCGTDKC NN

**Figure 3:**
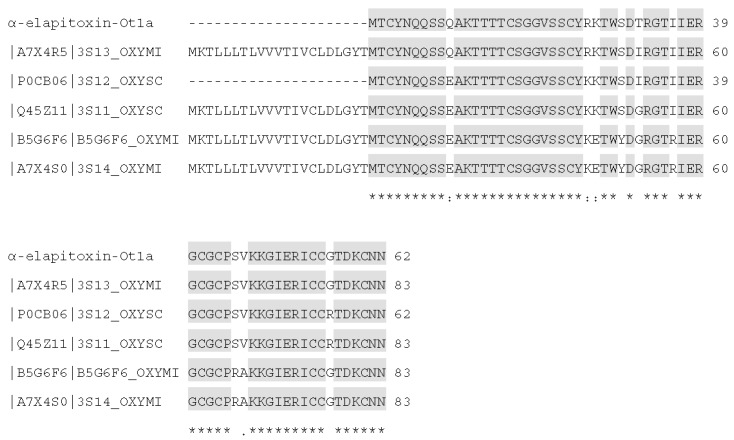
Sequence alignment (from BLAST search) of α-elapitoxin-Ot1a with short-chain postsynaptic neurotoxins from *Oxyuranus* spp. Shaded amino acids are similar to α-elapitoxin-Ot1a. Amino acids with (*) are fully conserved in all toxins, conserved amino acids with (.) are weakly similar properties group and amino acids with (:) are strongly similar properties group.

This sequence was identified to have the highest signal by ESI-LCMS/MS and *de novo* sequencing. Three other isoforms of this short-chain postsynaptic neurotoxin were also detected, however only partial sequences could be detected (data not shown). α-Elapitoxin-Ot1a showed a high degree of sequence similarity with short-chain postsynaptic neurotoxins from other taipan species (>87%) (Figure 3 and Table 1).

### 2.4. In Vitro Neurotoxicity

**Figure 4:**
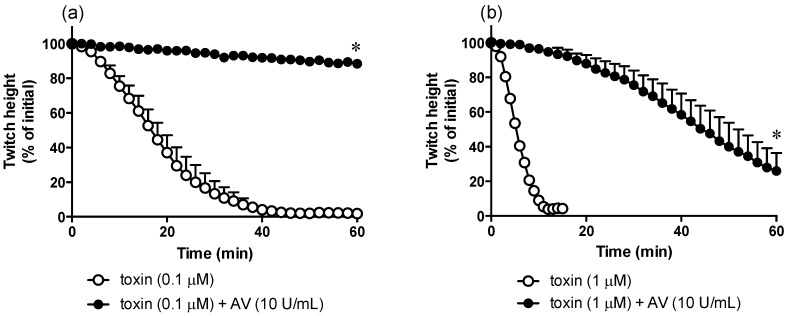
Effect of (**a**) α-elapitoxin-Ot1a (0.1 µM) alone and in the presence of taipan antivenom (AV; 10 U/mL) or (**b**) α-elapitoxin-Ot1a (1 µM) alone and in the presence of taipan antivenom (AV; 10 U/mL) on indirect twitches of the chick biventer cervicis nerve-muscle preparation. * *p* < 0.05, unpaired *t*-test, compared to α-elapitoxin-Ot1a alone at 60 min time point, *n* = 3.

**Figure 5:**
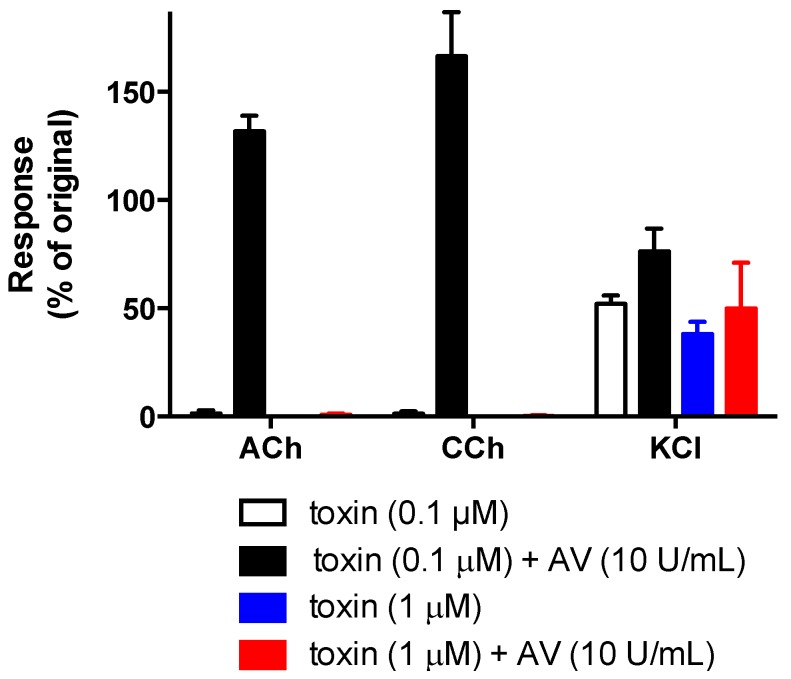
Effect of α-elapitoxin-Ot1a (0.1 µM or 1 µM) alone and in the presence of taipan antivenom (AV; 10 U/mL) on contractile responses of the chick biventer cervicis nerve-muscle preparation to acetylcholine (ACh; 1 mM), carbachol (CCh; 20 µM) and potassium chloride (KCl; 40 mM).

α-Elapitoxin-Ot1a (0.1 µM and 1 µM) caused concentration-dependent inhibition of twitches in the indirectly-stimulated chick biventer preparation (*n* = 3, Figure 4). At 1 µM, α-elapitoxin-Ot1a was strongly neurotoxic with a *t*_90_ value of 9.8 ± 0.4 min (Table 2). α-Elapitoxin-Ot1a also abolished contractile responses to exogenous ACh and CCh while only reducing KCl responses by approximately 50% (Figure 5).

Washing the tissue, at 10 min intervals commencing at the t_90_ time point (*i.e.*, time taken to produce 90% inhibition of nerve-mediated twitches) after the addition of α-elapitoxin-Ot1a (1 µM), did not produce any substantial recovery of twitch height even 3.5 h after the addition of α-elapitoxin-Ot1a (*n* = 3, data not shown). Preliminary data, where the concentration of α-elapitoxin-Ot1a was ten-fold less (*i.e.*, 0.1 µM) and a longer time of washing out was conducted did show that there was some degree of reversibility in the chick biventer cervicis nerve-muscle preparation, *i.e.*, recovery of around 33% after 280 min (4 h and 40 min) after toxin addition to tissue (*n* = 2, data not shown). These results suggest that the neurotoxic effects of α-elapitoxin-Ot1a are poorly reversible *in vitro*.

The prior addition of taipan antivenom (10 U/mL) almost abolished the neurotoxic actions of α-elapitoxin-Ot1a (0.1 µM) (Figure 4a) and significantly delayed the neurotoxic actions of α-elapitoxin-Ot1a (1 µM) (Figure 4b).

### 2.5. CCh Cumulative Concentration-Response Curves

**Figure 6:**
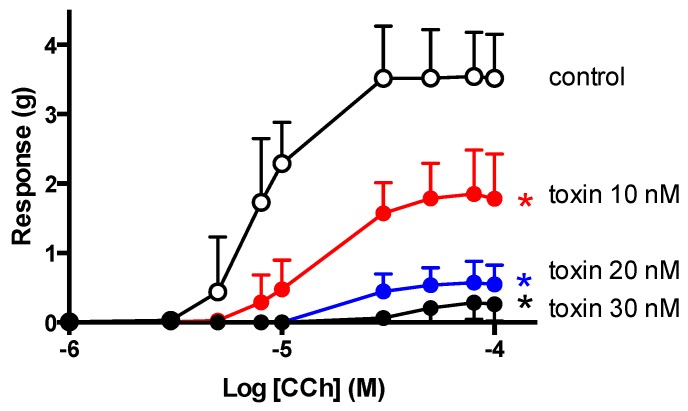
Effect of increasing concentrations of α-elapitoxin-Ot1a (10–30 nM; *n* = 3–4) on cumulative concentration-response curves to carbachol (CCh) in the chick biventer cervicis nerve-muscle preparation. * *p* < 0.05, significantly different from control, one-way ANOVA followed by Bonferroni's multiple comparisons test.

In unstimulated chick biventer preparations, α-elapitoxin-Ot1a (10–30 nM) induced concentration-dependent non-parallel shifts with a marked depression of maximum response to CCh (Figure 6). This indicates that α-elapitoxin-Ot1a is pseudo-irreversible in action. The modified Lew and Angus method was used to determine a pA_2_ value of 8.02 ± 0.05 for α-elapitoxin-Ot1a (Table 3).

## 3. Discussion

Snake venoms are comprised of numerous toxic and non-toxic components. The toxic components often include neurotoxins which assist in the immobilization and capture of prey but also may cause clinical neurotoxicity following systemic envenoming in humans. We have recently studied *O. temporalis* venom and shown that the reverse-phase HPLC venom profile consists of only a few peaks with the major peak eluting around 15 min [4]. Previous studies conducted in our laboratory have shown that, under the same reverse-phase HPLC conditions, short-chain postsynaptic neurotoxins elute from the reverse phase column between 15–17 min [16,17,21,22]. Therefore, *O. temporalis* venom was investigated further in order to isolate and characterize a short-chain postsynaptic neurotoxin from the venom.

**Figure 7:**
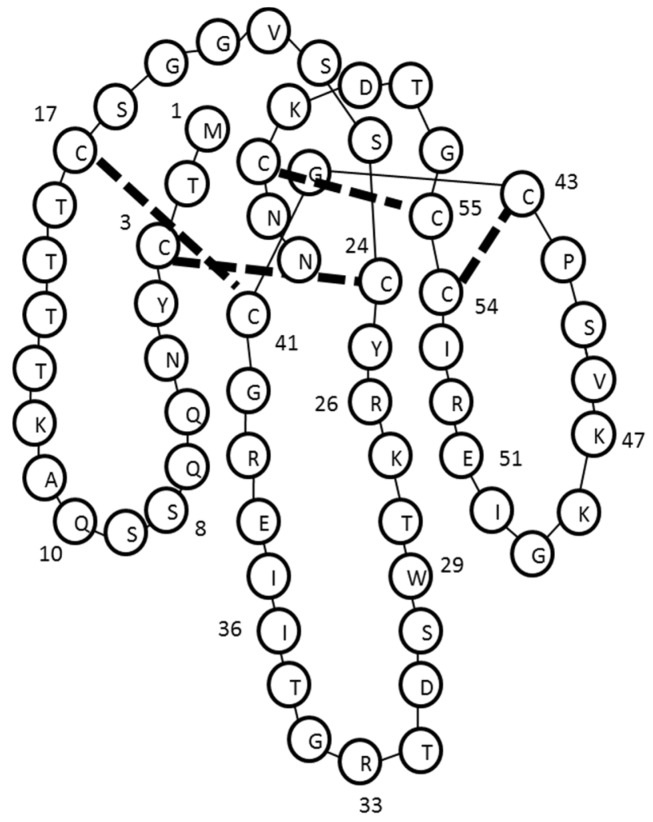
Structure of α-elapitoxin Ot1a based on the structure of erabutoxin B [24].

α-Elapitoxin-Ot1a, the first neurotoxin isolated from *O. temporalis* venom, was found to comprise 30.1% of the whole venom. Previous studies have shown that short-chain postsynaptic neurotoxins isolated from other taipan venoms represent between 1.1% (*i.e.*, taipan toxin 2; [18]) to 9% of the venom (*i.e.*, α-oxytoxin 1; [17]). Therefore, α-elapitoxin-Ot1a represents a far greater proportion of *O. temporalis* venom than the short-chain neurotoxins isolated from other taipan venoms. Typically short-chain post-synaptic neurotoxins have molecular masses between 6 and 7 kDa, while long-chain post-synaptic neurotoxins have larger molecular masses of between 7 and 9 kDa [19]. α-Elapitoxin-Ot1a, with a molecular mass of 6712 Da, falls within the range of the short-chain neurotoxins. *De novo* sequencing of the 15 min reverse-phase HPLC fraction revealed a structural profile indicative of short-chain postsynaptic neurotoxins (*i.e.*, the presence of eight cysteine residues in identical positions to previously isolated short-chain postsynaptic neurotoxins, Figure 7) (as previously reviewed [19]).

α-Elapitoxin-Ot1a shares a high degree of sequence similarity with previously identified taipan short-chain postsynaptic neurotoxins as well as taipan short-chain postsynaptic neurotoxins that have only been partially sequenced (*i.e.*, α-scutoxin 1; [17]), α-oxytoxin 1; [17]), oxylepitoxin 1; [16]). However, compared to short-chain postsynaptic neurotoxins from other seas snakes and terrestrial elapids, α-elapitoxin-Ot1a has much lower sequence identity, *i.e.*, 63% compared to erabutoxin A from *Laticauda semifasciata* venom and 73% compared to α-elapitoxin-Pc1a from *P. colletti* venom. *De novo* sequencing also indicated the presence of three other isoforms of α-elapitoxin-Ot1a. Unfortunately, only partial sequences of these isoforms could be detected and due to their similar amino acid sequences separation of these isoforms was not possible. Regardless, the combination of all four isoforms of α-elapitoxin-Ot1a represents a substantial proportion of *O. temporalis* venom (*i.e.*, approximately 30%).

The chick biventer cervicis nerve-muscle preparation, which contains both focally- and multiply-innervated muscle fibres, represents a simple and effective method of examining the neurotoxic activity of venoms and isolated components [25]. In this preparation, α-elapitoxin-Ot1a abolished nerve-mediated twitches much more rapidly than other elapid short-chain neurotoxins (Table 2). In a previous study, the short-chain neurotoxins α-oxytoxin 1 (from the previously named *O. s. canni* venom) and α-scutoxin 1 (from *O. scutellatus* venom) inhibited indirect twitches by 90% (*t*_90_ value) in approximately 25 and 12 min, respectively [17]. While oxylepitoxin-1 (from *O. microlepidotus* venom) was shown to have a *t*_90_ of approximately 55 min [16]. It should be noted that these experiments were carried out in an avian preparation (*i.e.*, chick biventer cervicis nerve-muscle preparation) and the potency of α-elapitoxin-Ot1a may differ in mammalian preparations as has been previously observed for other α-neurotoxins [20].

α-Elapitoxin-Ot1a also abolished contractile responses to nicotinic receptor agonists, as do other taipan short-chain postsynaptic neurotoxins [16,17], indicating that α-elapitoxin-Ot1a has a postsynaptic mode of action. In addition, the inhibitory effects of α-elapitoxin-Ot1a on nerve-mediated twitches were irreversible by washing. This is similar to hostoxin-1 [22], but in contrast to other taipan postsynaptic neurotoxins, *i.e.*, α-oxytoxin 1 [17] and oxylepitoxin-1 [16], which were reversible by washing of the tissue.

Cumulative concentration-response curves to CCh, in the absence and presence of increasing concentrations of α-elapitoxin-Ot1a, resulted in a concentration-dependent decrease in the maximal response to CCh with no parallel shift in the cumulative concentration response curve to CCh, indicating a pseudo-irreversible interaction of α-elapitoxin-Ot1a with skeletal muscle nicotinic acetylcholine receptors (nAChRs). That is, the toxin dissociates from the nAChR at such a slow rate that re equilibration is not achieved within the time frame of the experiment. Similarly, α-scutoxin 1 showed pseudo-irreversible activity in the avian preparation, while α-oxytoxin 1 was found to be reversible, as evidenced by a rightward parallel shift of the cumulative concentration-response curves to CCh with no depression of the maximal response [17]. Using the modified Lew Angus method of analysis, the pA_2_ value for α-elapitoxin-Ot1a was calculated to be 8.02 ± 0.05, which is approximately 5× less potent that α-bungarotoxin but 186× more potent than d-tubocurarine [23] (Table 3).

Although the clinical effects of envenoming by *O. temporalis* are unknown, it was important to elucidate whether the commercially available CSL taipan antivenom, which is raised against the venom of *O. scutellatus*, offers protection against the neurotoxic effects of α-elapitoxin-Ot1a. The *in vitro* inhibitory effects of α-elapitoxin-Ot1a were abolished or markedly delayed, depending on the concentration of toxin tested, indicating that the antivenom was effective under these conditions. Interestingly, in a previous study, taipan antivenom had little effect on the *in vitro* neurotoxic effects of oxylepitoxin-1 [16] while, unfortunately, the effect of antivenom against the taipan short-chain neurotoxins α-scutoxin 1 and α-oxytoxin 1 was not investigated [17]. However, this type of *in vitro* data should be interpreted with caution given the added complexities of treating envenomed humans with antivenom, and the often considerable delay between envenoming and the administration of antivenom.

Although it is clear that *O. temporalis* venom contains a high proportion of the short-chain postsynaptic neurotoxin α-elapitoxin-Ot1a (plus three other isoforms), whether or not this neurotoxin is able to bind at human nAChRs is unknown. Hart *et al.* [26] showed that *Pseudechis colletti* venom, which was later shown to contain mostly/only short-chain postsynaptic neurotoxins [20], was far less effective at blocking the actions of α-bungarotoxin at the human nAChR than nAChRs at the chick biventer neuromuscular junction. Similar results have been observed with other short-chain postsynaptic neurotoxins, including the sea snake short-chain postsynaptic neurotoxin erabutoxin [27,28].

In conclusion, this study reports the isolation and characterization of α-elaptitoxin-Ot1a, the first component isolated from *O. temporalis* venom. α-Elaptitoxin-Ot1a is a potent, pseudo-irreversible short-chain postsynaptic neurotoxin, whose inhibitory actions, in *in vitro* skeletal muscle preparations, can be prevented or delayed with prior administration of CSL Taipan antivenom. α-Elaptitoxin-Ot1a represents 30.1% of *O. temporalis* venom and also shares high amino acid sequence similarity with many other short-chain postsynaptic neurotoxins from Australian elapids.

## 4. Materials and Methods

### 4.1. Venom Supply

*O. temporalis* venom was obtained as previously described [4] from two live specimens of *O. temporalis* held at the Adelaide Zoo (Adelaide, South Australia, Australia). After extraction, the venom was frozen with dry ice, transferred into a −20 °C freezer and then lyophilized. After lyophilization, the venom was stored at 4–8 °C until required. The venom samples were then pooled, reconstituted with 0.15 M NaCl, aliquoted, re-frozen and lyophilized.

### 4.2. Purification α-Elapitoxin-Ot1a

All chromatography separations were performed using a high-performance liquid chromatography (HPLC) system (Shimadzu, Kyoto, Japan).

Freeze-dried *O. temporalis* venom was dissolved in Milli-Q water (Millipore Corporation, Billerica, MA, USA) to give a stock solution of 10 mg/mL. Samples were briefly vortexed, centrifuged at 14,000 rpm for 2 min and then applied to a Phenomenex (Phenomenex, Torrance, CA, USA) Jupiter semi-preparative (5 µm C18 300Å, 250 mm × 10 mm) C18 column that was equilibrated with 95% solvent A (0.1% trifluoroacetic acid, TFA) and 5% solvent B (90% acetonitrile (ACN) in 0.09% TFA). The samples were then eluted with the following gradient conditions of solvent B at a flow rate of 2 mL/min; 0%–20% B over 5 min, 20%–60% B between 5–40 min, 60%–80% B between 40–45 min, and finally 80%–0% B between 45–50 min. The eluent was monitored at 214 nm. Individual peaks were collected manually, frozen at −80 °C and then subsequently freeze-dried. α-Elapitoxin-Ot1a (*i.e.*, the peak at 15 min from the semi preparative reverse-phase HPLC) was further purified on a Phenomenex Jupiter analytical (150 mm × 2 mm, 5 µm, 300 Å) C18 after equilibrating with solvent A (0.1% TFA). The sample was then eluted using the following gradient conditions of solvent B (90% ACN in 0.09% TFA) at a flow rate 0.2 mL/min: 0%–20% over 0–5 min, 20%–60% in between 5 and 40 min and then 60%–80% over 40–45 min. The eluent was monitored at 214 nm. Purified α-elapitoxin-Ot1a was collected, frozen at −80 °C and then subsequently freeze-dried ready for further analysis.

### 4.3. Mass Spectrometry and Amino Acid Sequencing

#### 4.3.1. Intact Protein Analysis Using Matrix Associated Laser Desorption Time of Flight (MALDI-TOF) Mass Spectrometry

Intact protein analysis of α-elapitoxin-Ot1a was made using a MALDI TOF/TOF 4700 Proteomics Analyzer (Applied Biosystems; Foster City, CA, USA) with results analyzed using 4000 Series Explorer version 3.0 software (Applied Biosystems; Foster City, CA, USA) with 15 point smoothing applied. Samples were mixed 1:1 with 10 mg/mL α-cyano-4-hydroxycinnamic acid matrix (Laser BioLabs, Sophia-Antipolis, Valbonne, France) in 50% Acetonitrile 0.1% TFA and spotted onto the MALDI target plate. Proteins were analyzed in linear mode with a mass range of 5 kDa to 120 kDa.

#### 4.3.2. In-Solution Digestion of Sample in Preparation for Electrospray-Ionization Coupled with Mass-Spectrometry/Mass Spectrometry (ESI-LCMS/MS)

Protein (200 ng of the reverse-phase HPLC fraction eluting at 15 min) was mixed with 25 µL of 100 mM ammonium bicarbonate, 25 µL of trifluroethanol and 1 µL of 200 mM dithiothretiol (DTT) before being briefly vortexed, centrifuged and incubated at 60 °C for 1 h. Iodoacetamide (4 µL of 200 mM) was added and left in the dark at room temperature for 1 h. DTT (1 µL) was added and left to incubate at room temperature for another 1 h. Samples were diluted with Milli-Q grade water and ammonium bicarbonate to achieve pH 7–9. Trypsin was added based on a ratio of 1:20 (enzyme:protein) and incubated overnight at 37 °C. Formic acid (1 µL) was added at the end of the incubation to stop enzyme activity. The samples were dried in a vacuum concentrator and stored at −20 °C prior to analysis. Finally, 10 µL of 0.1% formic acid was added to the dried sample before it was loaded into an ESI-LCMS/MS system.

#### 4.3.3. Nanoflow Liquid Chromatography ESI-LCMS/MS

The digested sample was loaded into a Agilent C18 300 Å Large Capacity Chip (Agilent Technologies, Santa Clara, CA, USA). The column was equilibrated by 0.1% formic acid in MilliQ water (solution A) and peptides were eluted with an increasing gradient of 90% ACN in 0.1% formic acid (solution B) by the following gradient; 3%–50% solution B from 0–30 min, 50%–95% solution B from 30–32 min, 95% solution B from 32–39 min and 95%–3% solution B from 39–47 min. The polarity of the Q-TOF was set at positive, capillary voltage at 2150 V, fragmentor voltage at 300 V drying gas flow of 5 L/min and gas temperature of 300 °C. The spectrum was scanned in auto MS/MS mode over a range of 110–3000 *m/z* for MS scan and 50–3000 *m/z* range for MS/MS scan. The spectrum was analyzed by using Agilent MassHunter data acquisition software (Agilent Technologies, Santa Clara, CA, USA).

#### 4.3.4. Protein Identification by Automated *de Novo* Sequencing

*De novo* sequencing was conducted by using PEAKS Studio (Version 7.0, Bioinformatics Solution, Waterloo, ON, Canada, 2014). The identity search and *de novo* sequencing were performed by comparing *de novo* sequence tags with NCBInr Serpentes database (version April 2014, National Center for Biotechnology Information, Rockville Pike, MD, USA, 2014) in PEAKS DB and SPIDER modes. Carbamidomethylation was set as fixed modification and maximum missed cleavage was set at three. Parent mass and precursor mass tolerance was set at 0.1 Da. By using false detection rate (FDR) < 0.1% and −10log protein (−10logP) > 60, identified proteins were accepted if they have at least two peptides detected and one unique peptide. Maximum variable post-translational modification was set at four.

### 4.4. Neurotoxicity Studies

To examine the reversibility of the toxin, α-Elapitoxin-Ot1a (1 µM) was added to the organ baths and left in contact with the preparation until the t_90_ time point was reached (*i.e.*, the time at which 90% inhibition of the pre-venom/toxin twitch height was achieved). The tissue was then washed at 10 min intervals.

For studies examining the effectiveness of antivenom, tissue preparations were set up as above. Taipan antivenom (3 or 10 U/mL) was added to the organ bath 10 min prior to the addition of α-elapitoxin-Ot1a (0.1 µM or 1 µM).

### 4.5. CCh Cumulative Concentration-Response Curves

In order to determine the potency (*i.e.*, pA_2_ value) of α-elapitoxin-Ot1a, cumulative concentration–response curves to CCh were obtained in unstimulated chick preparations. Tissues were set up at 1 g tension on metal ring holders, allowed to equilibrate for 20 min, then α-elapitoxin-Ot1a (10–30 nM) or vehicle (Milli-Q water) was added to separate organ baths and left in contact with the tissue for 1 h. Following this incubation period, cumulative concentration-response curves to CCh were obtained in the presence of toxin or vehicle.

### 4.6. Data Analysis

The percentage of α-elapitoxin-Ot1a was determined from area under the curve analysis of the whole venom semi-preparative reverse-phase HPLC chromatograms. The area under curve for the peak representing α-elapitoxin-Ot1a (and its isoforms) was expressed as a percentage of the area under the curve for all peaks in the whole venom chromatogram.

For neurotoxicity experiments, twitch height was measured at regular time intervals and was expressed as a percentage of the pre-toxin twitch height. Contractile responses to ACh, CCh and KCl were expressed as a percentage of original responses. The time taken for 90% inhibition of the twitch response (*t*_90_ values) was used to compare the potency of α-elapitoxin-Ot1a with other elapid postsynaptic neurotoxins.

In order to determine antagonist potency (*i.e.*, pA_2_), cumulative concentration-response curves to CCh, obtained in the chick biventer, in the absence or presence of α-elapitoxin-Ot1a, were analyzed using the modified Lew Angus method (PRISM 6.0 GraphPad Software, San Diego, CA, USA, 2014).

For antivenom studies, an unpaired t test was used to analyze whether there was statistical significant difference (* *p* < 0.05) between α-elapitoxin-Ot1a alone and α-elapitoxin-Ot1a in the presence of taipan antivenom at the end of a 60 min time period.

### 4.7. Chemicals and Drugs

The following chemicals and drugs were used: acetylcholine chloride (Sigma-Aldrich, St. Louis, MO, USA), acetonitrile (ACN, Merck, Darmstadt, Germany), ammonium bicarbonate (Sigma-Aldrich), carbamylcholine chloride (carbachol; Sigma-Aldrich), dithiothretiol (Merck), formic acid (Sigma-Aldrich), iodoacetamide (GE Healthcare, Uppsala, Sweden), LCMS grade acetonitrile (Fisher Scientific, Loughborough, UK), potassium chloride (KCl, Ajax Finechem Pty. Ltd., Taren Point, Australia), proteomics grade bovine trypsin (Sigma-Aldrich), taipan antivenom (CSL Ltd., Melbourne, Australia), trifluoroacetic acid (TFA, Auspep, Melbourne, Australia), d-tubocurarine (Sigma-Aldrich) and trifluroethanol (Sigma-Aldrich). All chemicals were dissolved or diluted in MilliQ water for unless otherwise stated.

## Figures and Tables

**Table 1:** Partial or Full *N*-terminal sequence and molecular mass (Da) of α-elapitoxin-Ot1a in comparison to some other Australian elapid postsynaptic neurotoxins.

Species	α-Neurotoxin	MW	Partial/Full *N*-Terminal Sequence
*O. temporalis*	α-elapitoxin-Ot1a ^a^	6712	MTCYNQQSSQ AKTTTTCSGG VSSCYRKTWS DTRGTIIERG CGCPSVKKGI ERICCGTDKC NN
*O. scutellatus*	α-scutoxin 1 ^b^	6781	MTCYNQQSSE AKTTTTCSGG VSSCYKKTWY DGRGTRIERG
*O. scutellatus*	α-oxytoxin 1 ^b^	6770	MTCYNQQSSE AKTTTTCSGG VSSCYKETWY DGRGTT
*O. microlepidotus*	oxylepitoxin-1 ^c^	6789	MTCYNQQSSE AKTTTTCSGG VSSCYKETWY
*O. scutellatus*	Taipan toxin 1 ^d^	6726	MTCYNQQSSE AKTTTTCSGG VSSCYKKTWS DGRGTIIERG CGCPSVKKGI ERICCRTDKC NN
*O. scutellatus*	Taipan toxin 2 ^d^	6781	MTCYNQQSSE AKTTTTCSGG VSSCYKKTWS DIRGTIIERG CGCPSVKKGI ERICCRTDKC NN
*P. colletti*	α-elapitoxin-Pc1a ^e^	6759.6	MTCCNQQSSQ PKTTTTCAGG ETSCYKKTWS DHRGSRTERG CGCPHVKPGI KLTCCKTDEC NN
*P. porphyriacus*	α-elapitoxin-Ppr1 ^e^	6746.5	MTCCNQQSSQ PKTTTTCAGG ESSCYKKTWS DHRGSRTERG CGCPHVKPGI KLTCCETDEC NN
*P. papuanus*	Papuantoxin-1 ^f^	6738	MTCCNQQSSQ PKTTTT
*H. stephensi*	Hostoxin-1 ^g^	6660	MTPCNQQSSQ PKTTK

Note: Underlined amino acid residues have been deduced from the sequences of other short chain α-neurotoxins; ^a^ current study; ^b^ [17]; ^c^ [16]; ^d^ [18]; ^e^ [20]; ^f^ [21]; ^g^ [22].

**Table 2:** *t*_90_ values for elapid postsynaptic neurotoxins in chick biventer cervicis preparation.

Species	Common Name	Postsynaptic Neurotoxin	*t*_90_ (min) at 1 µM
*O. temporalis*	Western Desert Taipan	α-elapitoxin-Ot1a	9.83 ± 0.36 ^a^
*O. scutellatus*	Coastal Taipan	α-scutoxin 1	~12 ^b^
*O. scutellatus*	Papuan Taipan	α-oxytoxin 1	~25 ^b^
*O. microlepidotus*	Inland Taipan	oxylepitoxin-1	~55 ^c^
*H. stephensi*	Stephen’s Banded snake	hostoxin-1	~10 ^d^
*A.* sp. Seram	Seram death adder	Acantoxin IVa	9.7 ± 1.1 ^e^

^a^ current study; ^b^ [17]; ^c^ [16]; ^d^ [22]; ^e^ [23].

**Table 3:** pA_2_ values for postsynaptic neurotoxins and tubocurarine.

Snake Species	Common Name	Toxin	pA_2_ Value
*Bungarus multicinctus*	Various kraits	α-bungarotoxin	8.71 ± 0.06 ^a^
*Hoplocephalus stephensi*	Stephen’s Banded snake	hostoxin-1	8.45 ± 0.32 ^b^
*Oxyuranus scutellatus*	Coastal Taipan	α-scutoxin 1	8.38 ± 0.59 ^c^
*Acanthophis* sp. Seram	Seram death adder	acantoxin Iva	8.36 ± 0.17 ^a^
*Oxyuranus temporalis*	Western Desert Taipan	α-elapitoxin-Ot1a	8.02 ± 0.05 *
*Oxyuranus scutellatus*	Papuan Taipan	α-oxytoxin 1	7.62 ± 0.04 ^c^
*Oxyuranus microlepidotus*	Inland Taipan	oxylepitoxin-1	7.16 ± 0.28 ^d^
*Pseudechis colletti*	Collett’s snake	α -elapitoxin-Pc1	7.04 ± 0.07 ^e^
*Pseudechis prophyriacus*	Red bellied black snake	α-elapitoxin-Ppr1	6.97 ± 0.03 ^e^
*Pseudechis papuanus*	Papuan black snake	papuantoxin-1	6.90 ± 0.30 ^f^
Not applicable	-	d-tubocurarine	6.29 ± 0.06 ^a^

^a^ [23]; ^b^ [22]; ^c^ [17]; ^d^ [16]; ^e^ [20]; ^f^ [21]; * current study.

## Abbreviations

The following abbreviations are used in this manuscript:

ACh: acetylcholine
CCh: carbachol
HPLC: high performance liquid chromatography
ESI-LCMS/MS: electrospray-ionization coupled with mass-spectrometry/mass spectrometry

## References

[1] Doughty P., Maryan B., Donnellan S.C., Hutchinson M.N. (2007). A new species of taipan (Elapidae: *Oxyuranus*) from central Australia. Zootaxa.

[2] Shea G.M. (2007). A possible second record of the central ranges Taipan, *Oxyuranus temporalis* (Elapidae). Herpetofauna.

[3] Brennan K.E.C., Morley T., Hutchinson M., Donnellan S. (2012). Redescription of the western desert taipan, *Oxyuranus temporalis* (Serpentes: Elapidae), with notes on its distribution, diet and genetic variation. Aust. J. Zool..

[4] Barber C.M., Madaras F., Turnbull R.K., Morley T., Dunstan N., Allen L., Kuchel T., Mirtschin P., Hodgson W.C. (2014). Comparative studies of the venom of a new taipan species, *Oxyuranus temporalis*, with other members of its genus. Toxins.

[5] Fry B.G., Wickramaratana J.C., Lemme S., Beuve A., Garbers D., Hodgson W.C., Alewood P. (2005). Novel natriuretic peptides from the venom of the inland taipan (*Oxyuranus microlepidotus*): Isolation, chemical and biological characterisation. Biochem. Biophys. Res. Commun..

[6] Herrera M., Ferandez J., Vargas M., Villalta M., Segura A., Leon G., Angulo Y., Paiva O., Matainaho T., Jensen S.D. (2012). Comparative proteomic analysis of the venom of the taipan snake, *Oxyuranus scutellatus*, from Papua New Guinea and Australia: Role of neurotoxic and procoagulant effects in venom toxicity. J. Proteom..

[7] Speijer H., Govers-Riemslag J.W.P., Zwaal R.F.A., Rosing J. (1986). Prothrombin activation by an activator from the venom of *Oxyuranus scutellatus* (Taipan snake). J. Biol. Chem..

[8] Walker F.J., Owen W.G., Esmon C.T. (1980). Characterization of the prothrombin activator from the venom of *Oxyuranus scutellatus scutellatus* (Taipan venom). Biochemistry.

[9] Welton R.E., Burnell J.N. (2005). Full length nucleotide sequence of a Factor V-like subunit of oscutarin from *Oxyuranus scutellatus scutellatus* (coastal Taipan). Toxicon.

[10] Possani L.D., Martin B.M., Yatani A., Mochca-Morales J., Zamudio F.Z., Gurrola G.B., Brown A.M. (1992). Isolation and physiological characterization of taicatoxin, a complex toxin with specific effects on calcium channels. Toxicon.

[11] Earl S.T.H., Richards R., Johnson L.A., Flight S., Anderson S., Liao A., de Jersey J., Masci P.P., Lavin M.F. (2012). Identification and characterisation of Kunitz-type plasma kallikrein inhibitors unique to *Oxyuranus* sp. snake venoms. Biochimie.

[12] Fohlman J. (1979). Comparison of two highly toxic Australian snake venoms: The taipan (*Oxyuranus s. scutellatus*) and the fierce snake (*Parademansia microlepidotus*). Toxicon.

[13] Fohlman J., Eaker D., Karlsson E., Thesleff S. (1976). Taipoxin, an extremely potent presynaptic neurotoxin from the venom of the Australian snake taipan (*Oxyuranus s. scutellatus*). Isolation, characterization, quaternary structure and pharmacological properties. Eur. J. Biochem..

[14] Kuruppu S., Reeve S., Banerjee Y., Kini R.M., Smith A.I., Hodgson W.C. (2005). Isolation and pharmacological characterization of cannitoxin, a presynaptic neurotoxin from the venom of the Papuan Taipan (*Oxyuranus scutellatus canni*). J. Pharmacol. Exp. Ther..

[15] Barber C.M., Isbister G.K., Hodgson W.C. (2012). Solving the “Brown snake paradox”: *In vitro* characterisation of Australasian snake presynaptic neurotoxin activity. Toxicol. Lett..

[16] Clarke C., Kuruppu S., Reeve S., Smith A.I., Hodgson W.C. (2006). Oxylepitoxin-1, a reversible neurotoxin from the venom of the inland taipan (*Oxyuranus microlepidotus*). Peptides.

[17] Kornhauser R., Hart A.J., Reeve S., Smith A.I., Fry B.G., Hodgson W.C. (2010). Variations in the pharmacological profile of post-synaptic neurotoxins isolated from the venoms of the Papuan (*Oxyuranus scutellatus canni*) and coastal (*Oxyuranus scutellatus scutellatus*) taipans. Neuro Toxicol..

[18] Zamudio F., Wolf K.M., Martin B.M., Possani L.D., Chiappinelli V.A. (1996). Two novel α-neurotoxins isolated from the taipan snake, *Oxyuranus scutellatus*, exhibit reduced affinity for nicotinic acetylcholine receptors in brain and skeletal muscle. Biochemistry.

[19] Barber C.M., Isbister G.K., Hodgson W.C. (2013). Alpha neurotoxins. Toxicon.

[20] Hart A., Isbister G.K., O’Donnell P., Williamson N.A., Hodgson W.C. (2013). Species differences in the neuromuscular activity of post-synaptic neurotoxins from two Australian black snakes (*Pseudechis porphyriacus* and *Pseudechis colletti*). Toxicol. Lett..

[21] Kuruppu S., Reeve S., Smith A.I., Hodgson W.C. (2005). Isolation and pharmacological characterisation of papuantoxin-1, a postsynaptic neurotoxin from the venom of the Papuan black snake (*Pseudechis papuanus*). Biochem. Pharmacol..

[22] Tan L.C., Kuruppu S., Smith A.I., Reeve S., Hodgson W.C. (2006). Isolation and pharmacological characterisation of hostoxin-1, a postsynaptic neurotoxin from the venom of the Stephen’s banded snake (*Hoplocephalus stephensi*). Neuropharmacology.

[23] Wickramaratna J.C., Fry B.G., Loiacono R.E., Aguilar M.I., Alewood P.F., Hodgson W.C. (2004). Isolation and characterization at cholinergic nicotinic receptors of a neurotoxin from the venom of the *Acanthophis* sp. Seram death adder. Biochem. Pharmacol..

[24] Kimball M.R., Sato A., Richardson J.S., Rosen L.S., Low B.W. (1979). Molecular conformation of erabutoxin B; atomic coordinates at 2.5 A resolution. Biochem. Biophys. Res. Commun..

[25] Hodgson W.C., Wickramaratna J.C. (2002). *In vitro* neuromuscular activity of snake venoms. Clin. Exp. Pharmacol. Physiol..

[26] Hart A., Scott-Davey T., Harris J. (2008). Venom of Collett’s snake (*Pseudechis colletti*) blocks the binding of α-bungarotoxin to acetylcholine receptors at chick but not human neuromuscular junctions: A histochemical study. Toxicon.

[27] Ishikawa Y., Kano M., Tamiya N., Shimada Y. (1985). Acetylcholine receptors of human skeletal muscle: A species difference detected by snake neurotoxins. Brain Res..

[28] Vincent A., Jacobson L., Curran L. (1998). α-Bungarotoxin binding to human muscle acetylcholine receptor: Measurement of affinity, delineation of AChR subunit residues crucial to binding, and protection of AChR function by synthetic peptides. Neurochem. Int..

